# Wnts Promote Synaptic Assembly Through T-Cell Specific Transcription Factors in *Caenorhabditis elegans*

**DOI:** 10.3389/fnmol.2018.00194

**Published:** 2018-06-15

**Authors:** Yanjun Shi, Qian Li, Zhiyong Shao

**Affiliations:** Department of Neurology, State Key Laboratory of Medical Neurobiology and Institutes of Brain Science, Zhongshan Hospital, Fudan University, Shanghai, China

**Keywords:** synaptic assembly, CWN-2/Wnt, canonical Wnt pathway, CFZ-2/Frizzled, DSH-2/Dishevelled, SYS-1/β-catenin, POP-1/TCF/LEF, intestine-neuron cross talk

## Abstract

Synapses are specialized neuronal connections essential for neuronal function. Defects in synaptic assembly or maintenance usually lead to various neurological disorders. Synaptic assembly is regulated by secreted molecules such as Wnts. Wnts are a large family of conserved glycosylated signaling molecules involved in many aspects of neural development and maintenance. However, the molecular mechanisms by which Wnts regulate synaptic assembly remain elusive due to the large number of ligands/receptors, the diversity of signaling cascades and the complexity of the nervous system. In this study, through genetic manipulation, we uncover that *C. elegans* Wnt-2 (CWN-2) is required for synaptic development. The CWN-2 signal is required during both embryonic and postembryonic development, in the nervous system and intestine, for promoting synaptic assembly. Furthermore, we provide genetic evidence for CWN-2 promoting synaptogenesis through the Frizzled receptor (FZD) CFZ-2, the Dishevelled (DVL) DSH-2, the β-catenin SYS-1 and the only T-cell specific transcription factor POP-1/TCF. Importantly, it is the first time to report the requirement of a TCF for presynaptic assembly. These findings expand our understanding of the synaptogenic mechanisms and may provide therapeutic insights into Wnt-related neurological disorders.

## Introduction

Normal neural function requires precise synaptic connections, and defects in the connection often result in neurological disorders (Caracci et al., 2016; Remedio et al., 2016; Song et al., 2016, 2017; Roeper, 2017; Zhai et al., 2017; Moretto et al., 2018). Chemical synapses are junctional connections composed of presynaptic terminals, postsynaptic targets and the synaptic cleft (Pappas and Purpura, 1972; Cowan et al., 2001). Synaptic formation and maintenance are finely regulated by signaling molecules such as Wnts (Wu et al., 2010; Budnik and Salinas, 2011; Henríquez and Salinas, 2012; Park and Shen, 2012; Dickins and Salinas, 2013).

Wnts are a large family of conserved glycosylated secreted signaling molecules, with 19 members in mammals, seven in fly, and five in nematode (Gordon and Nusse, 2006; Willert and Nusse, 2012; Barik et al., 2014). Upon binding to their receptors, Wnts trigger conserved signaling cascades including the canonical β-catenin/TCF pathway and noncanonical planer cell polarity (PCP) and Ca^2+^ pathways (Supplementary Figure S1; Mlodzik, 1999; Patapoutian and Reichardt, 2000; Ciani and Salinas, 2005; Montcouquiol et al., 2006). In *Drosophila*, Wnts also act through the Frizzled (FZD) nuclear import signaling pathway (Mathew et al., 2005).

Wnts play complex roles at multiple levels in synaptic development due to the large number of ligands/receptors and the diversity of signal cascades. Wnts activate different signaling cascades to regulate synaptic assembly. For examples, Wnt7a promotes presynaptic assembly through the canonical pathway (Lucas and Salinas, 1997; Hall et al., 2000; Cerpa et al., 2008; Davis et al., 2008), while it promotes postsynaptic PSD95 expression or spine growth by non-canonical Ca^2+^ signal pathways (Ciani et al., 2011). The role of Wnts in synaptogenesis is conserved in metazoans (Packard et al., 2002; Inaki et al., 2007; Klassen and Shen, 2007; Jing et al., 2009; Jensen et al., 2012a; Park and Shen, 2012). Although it is well known that Wnts are required to regulate synaptic assembly, many questions remain. For example, systematic studies of Wnts in synaptic assembly are missing. Additionally, although the expression of T-cell specific transcription factor 1 (TCF1) and Lymphoid Enhancing Factor 1 (LEF1), the downstream components in the canonical Wnt pathway, was associated with memory consolidation in mice (Fortress et al., 2013), the requirement of TCF/LEF molecules for synaptic assembly or maintenance has not been reported in any system.

*C. elegans* has proven to be an excellent model for addressing molecular mechanisms underlying synaptogenesis *in vivo* at the single cellular level in live animals (Jin, 2005). Wnt signal pathways are conserved in the nematode *C. elegans* (Sawa and Korswagen, 2013), and regulate neuromuscular junction (NMJ) synaptic assembly and plasticity (Klassen and Shen, 2007; Jensen et al., 2012b; Mizumoto and Shen, 2013; Pandey et al., 2017). However, it remains unknown if Wnts are required for non-NMJ presynaptic formation in the nematode nerve ring, which is analogous to the vertebrate brain. To address this question, we systematically examined the requirement of all five Wnts, four Frizzled receptors (FZDs), three Dishevelled (DVLs), four β-catenin and only one POP-1/TCF for the presynaptic assembly in the Amphid interneurons (AIY). We found that genes encoding components in the canonical Wnt pathway, including *cwn-2/Wnt*, *cfz-2/Fzd*, *dsh-2/Dvl*, *sys-1/β-catenin* and the *pop-1/Tcf*, are required for promoting AIY synaptic assembly during both embryonic and postembryonic stages both in the nervous system and in the intestine.

## Materials and Methods

### Strains and Genetics

All worms were fed with *E. coli* OP50 on standard NGM plates as described (Brenner, 1974). Strains used in this study were maintained at 21°C and detailed information is listed in Supplementary Table S1. The mutants and transgenic alleles were used in this study: *wyls45* (*Pttx-3::GFP::rab-3, Punc-122::RFP*) X, *cwn-1(ok546)* II, *cwn-2(ok895)* IV, *lin-44(n1792)* I, *egl-20(n585)* IV, *mig-1(e1787)* I, *lin-18(e620)* X, *lin-17(n671)* I, *cfz-2(ok1201)* V, *dsh-1(ok1445)* II, *bar-1(mu63)* X, *cdc-42(ok825)* II, *vang-1(ok1142)* X, *pop-1(hu9)* I, *olais10*(*Pttx-3::mCherry::rab-3, Pttx-3::GFP::syd-1, Punc-122::RFP)*, *shcEx293* (*Pcwn-2::GFP, Punc-122::RFP*), *shcEx312*(*Pcwn-2::GFP, Punc-122::RFP*), *shcEx112*(*Pcwn-2::cwn-2, Punc-122::GFP*), *shcEx113*(*Pcwn-2::cwn-2, Punc-122::GFP*), *shcEx114* *(Pcwn-2::cwn-2, Punc-122::GFP*), *shcEx280*(*Prab-3::cwn-2, Punc-122::GFP*), *shcEx437*(*Prab-3::cwn-2, Punc-122::GFP*), *shcEx438(Prab-3::cwn-2, Punc-122::GFP*), *shcEx267*(*Pmyo-2::cwn-2, Punc-122::GFP*), *shcEx279*(*Pmyo-2::cwn-2, Phlh-17::mCherry*), *shcEx439*(*Pmyo-3::cwn-2, Punc-122::GFP*), *shcEx440*(*Pmyo-3::cwn-2, Punc-122::GFP*), *shcEx448*(*Pttx-3::cwn-2, Punc-122::GFP)*, *shcEx449*(*Pttx-3::cwn-2, Punc-122::GFP*), *shcEx450*(*Pges-1::cwn-2, Punc-122::GFP*), *shcEx451*(*Pges-1::cwn-2, Punc-122::GFP*), *shcEx*452(*Pges-1::cwn-2, Punc-122::GFP*), *shcEx484*(*Pcfz::cfz-2, Punc-122::GFP*), *shcEx*485 (*Pcfz::cfz-2, Punc-122::GFP*), *shcEx444*(*Pttx-3::cfz-2, Punc-122::GFP*), *shcEx445*(*Pttx-3::cfz-2, Punc-122::GFP*), *shcEx446*(*Pttx-3::cfz-2, Punc-122::GFP*), *shcEx453*(*Prab-3::cfz-2, Punc-122::GFP*), *shcEx454*(*Prab-3::cfz-2, Punc-122::GFP*), *shcEx455*(*Prab-3::cfz-2, Punc-122::GFP*), *shcEx479*(*Pges-1::cfz-2, Punc-122::GFP*), *shcEx480*(*Pges-1::cfz-2, Punc-122::GFP*), *shcEx481*(*Pges-1::cfz-2, Punc-122::GFP*), *shcEx482*(*Pmyo-2::cfz-2, Punc-122::GFP*), *shcEx483*(*Pmyo-2::cfz-2, Punc-122::GFP*), *ShcEx311(Pcfz-2::GFP), shcEx661(Pttx-3::mCherry, Pcfz-2::GFP), shcEx662(Pttx-3::mCherry, Pcwn-2::GFP), shcEx665(Pttx-3::mCherry), shcEx666(Pttx-3::mCherry), shcEx667(cfz-*2 *genomic::GFP, phlh-17::mCherry), shcEx668(cwn-*2 *genomic::GFP, phlh-17::mCherry)*.

### Cloning and Transgenes

We created the expression clones using the pSM vector, a derivative of pPD49.26 (A. Fire) with extra cloning sites (Shen and Bargmann, 2003) or the Gateway pDEST vector from Invitrogen. The *cwn-2* promoter was 2.6 kb sequence upstream from the start codon. The *cfz-2* promoter was 2.9 kb sequence upstream from the start codon. The *rab-3*, *ttx-3*, *ges-1*, *myo-2*, *myo-3* tissue-specific promoters were designed based on previous studies (McGhee et al., 1990; Okkema et al., 1993; Nonet et al., 1997; Wenick and Hobert, 2004). The *rab-3*, *ttx-3*, *ges-1*, *myo-2* and *myo-3* promoters were inserted into upstream of *cwn-2* or *cfz-2* genomic sequence. Why we use the genomic sequence instead of the cDNA is because the *cwn-2* cDNA driven by its own promoter did not rescue the mutant defect (data not shown). To examine if the genomic sequence has tissue-specific regulatory element, we drove GFP expression with genomic *cwn-2* or *cfz-2* and did not observe any GFP expression (Supplementary Figures S4K,L, S6E,F). RNA interference (RNAi) constructs were made by inserting corresponding cDNA, except for *sys-1*, which is the genomic sequence, into the double inverted T7 L4440 vector (pPD129.36). We utilized the standard microinjection techniques (Mello et al., 1991) to generate the transgenic strains. The genomic *cwn-2* fragment (P*cwn-2::cwn-2*) was injected at 5 ng/μl with *Punc-122::GFP* (20 ng/μl). All plasmids were injected at 20 ng/μl with coelomocyte (*Punc-122::GFP* or *Punc-122::mCherry*) or CEPsh glia (*Phlh-17::mCherry*) as a coinjection marker (20 ng/μl). The detail constructs information is listed in Supplementary Table S2.

### RNA Interference

RNAi constructs were transformed into HT115. The RNAi bacteria and plates were prepared as described previously (Fraser et al., 2000). For each RNAi treatment, we collected eggs from 10 synchronized day 1 adults for 2 h and fed them with RNAi bacteria. Then AIY synaptic phenotype was scored 66 h later when they reached the day 1 adult stage. To quantified the effect of *cwn-2* or *pop-1* knockdown from different developmental stages, synchronized L1, L2, L4, adult (day 1) animals (12, 20, 42, 66 h after eggs, respectively) were fed with RNAi bacteria and scored for the AIY presynaptic phenotype 3 days later. The *wrm-1, dsh-2, sys-1* and *pop-1* RNAi efficiency is also verified by quantifying the lethality (Supplementary Figure S9).

### Fluorescence Microscopy and Confocal Imaging

Animals were synchronized at specific stages. Larva and adult animals were immobilized using 50 mM muscimol on 3% agarose pad. For examining AIY Zone 2 morphology, we used the Nikon Ni-U fluorescent microscope with FITC filter and 40× objectives. All images presented in this study and for fluorescent intensity quantification were taken with Perkin Elmer UltraView VoX or Andor Dragonfly Spinning Disc Confocal Microscope with 40× objectives and 488 nm (for GFP) or 561 nm (for mCherry or RFP) laser. Images were displayed as extended focus projection. We used Adobe photoshop CC to process the rotation and brightness/contrast levels.

### Quantification

Wild type AIY Zone 2 synaptic structure forms a large cluster. The AIY Zone 2 presynaptic fragmentation defined as the AIY zone 2 becomes more than one smaller clusters. For each analysis, at least 20 synchronized animals were scored blindly for each genotype for at least three biological replicates. For rescue analysis, data were collected from three independent transgenic lines. L1, L2, L3, L4, adult (day 1) animals were synchronized as 12, 20, 30, 42, 66 h after eggs laid, respectively. We use the velocity to quantify the fluorescence intensity in AIY Zone 2 and Zone 3. Zone 2 was morphologically defined at the AIY elbow region where the ventral AIY neurite process enters the nerve ring with 10 micrometers in length. Zone 3 is from the distal site of Zone 2 to the tip of the AIY neurites. In the process of analyzing images, we draw a dashed frame figure 1.6 times as long as it wide with the corner as the symmetric center point.

### Statistical Analyses

We determined the *P* values using GraphPad Prism 6.0 (GraphPad Software). Statistical significance was analyzed using either *t*-students test or ANOVA as indicated in the figure legends.

## Results

### CWN-2/Wnt Is Required for AIY Presynaptic Clustering

**Figure 1:**
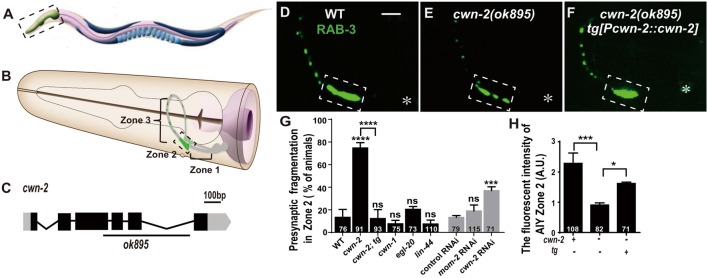
*cwn-2*/Wnt is required for presynaptic vesicle clustering.** (A)** Cartoon diagram of a nematode *C. elegans*.** (B)** The head region shown in the dashed box in **(A)**. Bilateral asymmetric Amphid interneurons (AIY) are indicated in gray, whose neurites innervate in the nerve ring. AIY presynapses form a distinct and highly reproducible pattern: the ventral region proximal to soma with no synapse (Zone 1), the elbow turn region with a large synaptic cluster (Zone 2) and the distal region with a few scattered synapses (Zone 3; Colón-Ramos et al., 2007; Altun et al., 2002–2018). **(C)** *cwn-2(ok895)* is a 905 bp deletion allele. The boxes and lines represent exons and introns of *cwn-2* gene. Black and gray indicate coding sequence and UTRs. The line beneath indicates the deletion region. **(D–F)** Confocal images of AIY synaptic marker GFP::RAB-3 in wild type **(D)**, *cwn-2(ok895)* mutants **(E)** and *cwn-2(ok895)* mutants rescued by a transgene (tg[*Pcwn-2::cwn-2*]) **(F)**. The Zone 2 of AIY forms a large presynaptic cluster in wild type animals, while the cluster is broken into multiple pieces, which is named fragmentation, in *cwn-2(ok895)* mutants. This defect is rescued by transforming a copy of wild type *cwn-2*. The dashed boxes indicate AIY Zone 2. Asterisks indicate AIY soma. Scale bars, 10 μm applied in all panels.** (G,H)** Quantification of AIY Zone 2 fragmentation **(G)** and fluorescence intensity **(H)**. The mutant alleles are *cwn-1(ok546), egl-20(n585), lin-44(n1792), cwn-2(ok895)*. tg: *Pcwn-2::cwn-2* transgene; +: wild type or with tg; −: mutant or without tg; ns: not significance, *p < 0.05, ***p < 0.001, ****p < 0.0001, analyzed by one-way analyses of variance (ANOVA) Dunnett’s test. Error bars represent 95% confidence interval.

*C. elegans* AIYs are a pair of bilateral symmetric interneurons located in the nerve ring (Figures 1A,B; White et al., 1986). AIY neurites can be divided into three Zones based on the anatomic location: the ventral part proximal to the soma, called Zone 1; the distal axon in nerve ring, called Zone 3; and the middle elbow region called Zone 2 (Figure 1B; White et al., 1986; Colón-Ramos et al., 2007). AIY forms large presynaptic clusters in Zone 2, which can be labeled with the synaptic vesicle marker GFP::RAB-3, and the synaptic clustering phenotype is highly reproducible across individual animals (87%, Figures 1D,G; Colón-Ramos et al., 2007).

To test whether Wnts are required for synaptic clustering, we examined the AIY synaptic vesicle marker GFP::RAB-3 clustering phenotype in all four viable Wnt loss of function mutants: *cwn-1(ok546), cwn-2(ok895), egl-20(n585), lin-44(n1792*; their genetic lesions are shown in Figure 1C, Supplementary Figures S2A–C), and in the essential Wnt *mom-2* knockdown animals. Among those mutant alleles we examined, *cwn-1(ok546)*, *cwn-2(ok895)* and *lin-44(n1792)* are most likely to be null alleles since the first two are big deletions and the third is an early stop (Herman et al., 1995; Zinovyeva and Forrester, 2005). The *egl-20(n585)* is probably a strong loss of function mutation since the altered a highly conserved cysteine at position 99 to a Serine (Maloof et al., 1999). We only found that *cwn-2* is required for the synaptic clustering as revealed by the synaptic vesicle GFP::RAB-3 marker (Figures 1E,G,H and Supplementary Figures S2F–J). In *cwn-2(ok895)* mutants, the coherent Zone 2 GFP::RAB-3 clustering is fragmented in 74.7% animals (*p* < 0.0001, Figures 1E,G). Additionally, we quantified the relative Green fluorescent protein (GFP) intensity and found that the intensity of GFP::RAB-3 is reduced by 60.2% (*p* < 0.001, Figures 1E,H). To confirm the requirement of *cwn-2* for AIY synaptic clustering, we knocked down *cwn-2* by RNAi and found robust AIY Zone 2 synaptic fragmentation in *cwn-2* RNAi treated animals as well (*P* < 0.001, Figure 1G). The requirement of *cwn-2* for GFP::RAB-3 clustering is further confirmed by the fact that the AIY synaptic defect in *cwn-2(ok895)* mutants can be rescued by expressing the wild type *cwn-2* transgene in *cwn-2(ok895)* mutants (*P* < 0.0001 for Zone 2 fragmentation, *P* < 0.05 for GFP intensity, Figures 1F–H). To examine if the AIY Zone 3 region is affected by *cwn-2(ok895)*, we quantified the GFP intensity and found that the GFP intensity in the AIY Zone 3 region is normal in *cwn-2(ok895)* mutants (Supplementary Figure S3).

**Figure 2:**
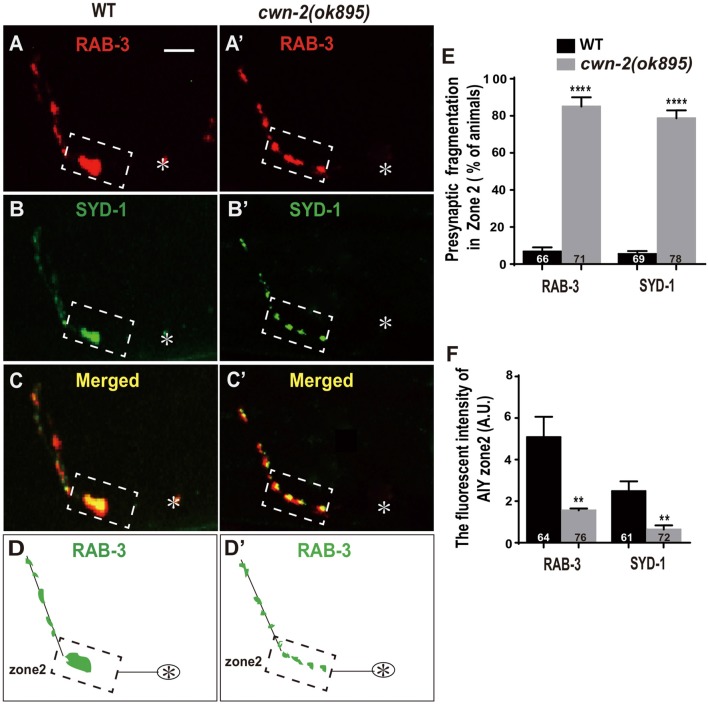
*cwn-2*/Wnt is required for presynaptic active zone clustering.** (A–C, A’–C’)** Confocal micrographs of the AIY synaptic vesicle marker mCherry::RAB-3 and synaptic active zone GFP::SYD-1 in wild type **(A,B)** and *cwn-2(ok895)* mutants **(A’,B’)**. Those two presynaptic markers are also colocalized, as indicated in the merged images **(C,C’)**. **(D,D’)** The schematic diagram of AIY presynaptic distribution in wild type** (D)** and *cwn-2(ok895)* mutants** (D’)**. The dashed boxes highlight the AIY Zone 2. Asterisks indicate AIY soma. Scale bars: 10 μm for all panels. **(E,F)** The quantitative data for Zone 2 fragmentation **(E)** and fluorescence intensity **(F)** for synaptic vesicle and active zone markers. **p < 0.01, ****p < 0.0001, analyzed by two-tailed Student’s *t*-test. Error bars represent standard errors of the mean (SEM).

Synaptic vesicle and synaptic active zone proteins are assembled independently (Zhen and Jin, 2004). To address if *cwn-2* is also required for AIY synaptic active zone protein assembly, we examined the synaptic active zone marker GFP::SYD-1 (Hallam et al., 2002). We found that GFP::SYD-1 colocalizes with the synaptic vesicle marker mCherry::RAB-3, as reported previously (Figures 2A–D; Stavoe and Colon-Ramos, 2012; Shao et al., 2013). Similar to the RAB-3 marker, the intensity of SYD-1::GFP is dramatically reduced and the Zone 2 GFP is fragmented in *cwn-2(ok895)* mutants (*P* < 0.0001 for Zone 2 fragmentation, *P* < 0.01 for fluorescent intensity, Figures 2A′–D′,E,F). Together, these data suggest that *cwn-2* is required for both AIY synaptic vesicle and active zone protein assembly in the Zone 2 region. Since SYD-1 marker and RAB-3 marker are colocalized and the presynaptic defect is the same for both markers in *cwn-2(ok895)*, we only use GFP::RAB-3 for our further analysis.

### CWN-2 Promotes Synaptogenesis During Both Embryonic and Postembryonic Development

**Figure 3:**
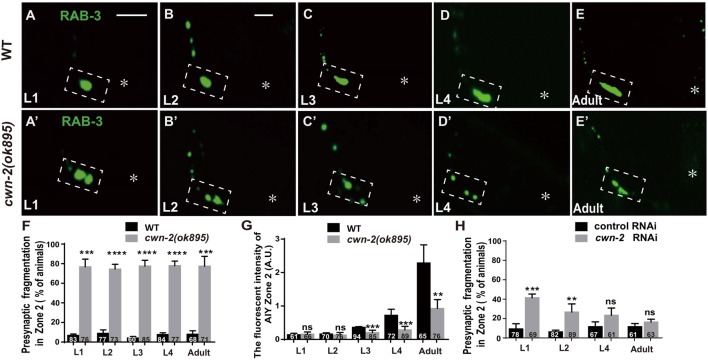
*cwn-2* is required for AIY synaptic clustering during embryonic and postembryonic development. **(A–E,A’–E’)** Confocal micrographs of the AIY presynaptic pattern of GFP::RAB-3 at different developmental stages in wild type **(A–E)** and *cwn-2(ok895)* mutant **(A’–E’)** animals. The dashed boxes highlight the AIY Zone 2. Asterisks indicate AIY soma. **(A,A’)** use the same scale, and the rest images use the same scale. Scale bars: 10 μm. **(F–H)** The quantitative data for Zone 2 fragmentation **(F,H)** and fluorescence intensity **(G)** for synaptic vesicle marker. The Zone 2 fragmentation appears beginning at newly hatched L1 and remains through adulthood **(F)**, while the fluorescence intensity is normal before L2 stage and significantly reduced beginning at L3 **(G)** in *cwn-2(ok895)* mutants. Postembryonic knockdown of *cwn-2* from L1 or L2, but not from L4 or adult stage results robust Zone 2 fragmentation **(H)**. Data are averaged from at least three biological replicates. ns: not significant, **p < 0.01, ***p < 0.001, ****p < 0.0001, analyzed by two-tailed Student’s *t*-test. Error bars represent SEM.

The previous described synaptic phenotype in *cwn-2(ok895)* could result from the defect of synaptic assembly during embryogenesis or synaptic maintenance during postembryonic stages. To differentiate those two, we examined the AIY synaptic marker GFP::RAB-3 at four larval stages (L1–L4) and the adult stage. In wild type animals, AIY forms a large synaptic cluster at Zone 2 region immediately upon hatching (6.8% abnormal, Figure 3A). The size of the GFP::RAB-3 cluster increases and the morphology remains consistent during growth (Figures 3A–G). However, in *cwn-2(ok895)* mutants, the Zone 2 fragmentation appears at the first larval stage L1 (76.8% abnormal) and continues into adulthood (Figures 3A′–E′,F). Additionally, although the GFP::RAB-3 intensity is similar between wild type and *cwn-2(ok895)* mutants from hatching to L2 stage (*P* = 0.64 at L1, *P* = 0.38 at L2), it is significantly reduced beginning in L3 stage in *cwn-2(ok895)* mutants (*P* < 0.001 at L3, *P* < 0.001 at L4, *P* < 0.01 at adult, Figures 3A′–E′,G). These results suggest that *cwn-2* is required not only for synaptic formation during embryonic development, but also for synaptic expansion during postnatal growth.

To confirm the requirement of *cwn-2* during the postembryonic development further, we treated wild type animals with *cwn-2* RNAi beginning at different stages (L1, L2, L4 and day 1 adult) and examined the AIY presynaptic phenotype 3 days later. We found that postembryonic knockdown of *cwn-2* from either L1 or L2, but not from L4 or adult day 1 stage, also led to a synaptic fragmentation in the AIY Zone 2 (*P* < 0.001 at L1, *P* < 0.01 at L2, Figure 3H).

### CWN-2 Acts Both in the Nervous System and in the Intestine to Regulate AIY Synaptic Clustering

To determine where *cwn-2* acts, we first determine the expression pattern by the transcription reporter P*cwn-2::GFP*. Consistent with previous findings, P*cwn-2::GFP* is expressed beginning in early embryonic stages, mainly in the digestive and nervous systems, with weak expression in the body wall muscles at adult stage (Supplementary Figure S4 and data not shown; Kennerdell et al., 2009; Song et al., 2010). The GFP reporter is only seen in the intestine before 2-fold stage (Supplementary Figures S4A–C). In late embryonic stage, P*cwn-2::GFP* is expressed both in the intestine and the pharynx (Supplementary Figures S4D,E). After hatching, P*cwn-2::GFP* is mainly seen in the pharynx, some head neurons, the body wall muscle and the intestine (Supplementary Figures S4F,G). However, P*cwn-2::GFP* is not seen in the AIY (Supplementary Figures S4H–J).

**Figure 4:**
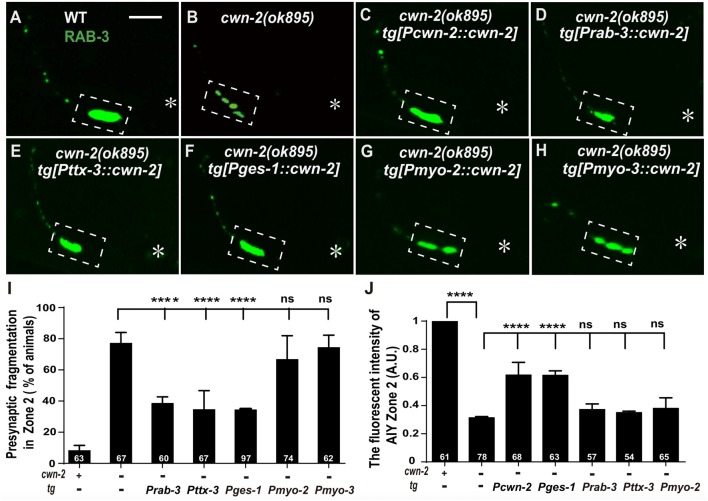
*cwn-2* acts in nervous system and intestine to regulate AIY synaptic clustering.** (A–H)** Confocal micrographs of the AIY presynaptic pattern of GFP::RAB-3. Presynaptic marker GFP::RAB-3 forms a large continuous cluster at AIY Zone 2 in wild type animals **(A)**, and the cluster is fragmented in *cwn-2(ok895)* mutants **(B)**. This Zone 2 fragmentation in *cwn-2(ok895)* is rescued by driving expression of *cwn-2* with its own promoter **(C)**, pan-neuronal *rab-3* promoter **(D)**, AIY-specific *ttx-3* promoter **(E)** or intestinal-specific *ges-1* promoter **(F)**, but not with pharyngeal-specific *myo-2* promoter **(G)** or body wall muscle-specific *myo-3* promoter **(H)**. Dashed boxes highlight AIY zone 2 and asterisks indicate the position of AIY soma. The scale bar represents 10 μm. **(I,J)** Quantification of the GFP::RAB-3 fragmentation **(I)** or fluorescence intensity **(J)** in AIY Zone 2. The Zone 2 fragmentation phenotype is rescued by expressing *cwn-2* either in the nerve system (with pan-neuronal *rab-3* promoter or with AIY-specific *ttx-3* promoter) or in the intestine (with *ges-1* promoter), while the GFP::RAB-3 intensity can only be rescued by expressing *cwn-2* in the intestine, not in the nerve system. Data for each genotype are averaged from at least three biological replicates. Transgenic data are averaged from at least two independent lines. ns: not significance, ****p < 0.0001, analyzed by one-way ANOVA Dunnett’s test. Error bars represent 95% confidence interval.

Given that *cwn-2* is mainly expressed in the nervous system, intestine, pharynx and body wall muscle, we next expressed *cwn-2* in those tissues with tissue-specific promoters P*rab-3* (neurons), P*ges-1*(intestine), P*myo-2* (pharynx) or P*myo-3* (muscles) in *cwn-2(ok895)* mutants. AIY Zone 2 fragmentation is rescued when *cwn-2* is expressed in the nervous system with the pan-neuronal *rab-3* promoter or the AIY-specific *ttx-3* promoter, or in the intestine with the *ges-1* promoter, but not in the pharynx or body wall muscle (*P* < 0.0001 for P*rab-3::cwn-2*, P*ttx-3*::*cwn-2* and P*ges-1::cwn-2*. Figures 4A–I). However, only intestinal expression of *cwn-2* rescues the GFP::RAB-3 intensity (Figure 4J). The data suggest that the presynaptic morphology is regulated by either the local neuronal or the distant intestinal CWN-2, while the presynaptic GFP::RAB-3 intensity is only regulated by the intestinal CWN-2.

### CFZ-2/FZD Is Required for AIY Synaptic Clustering

**Figure 5:**
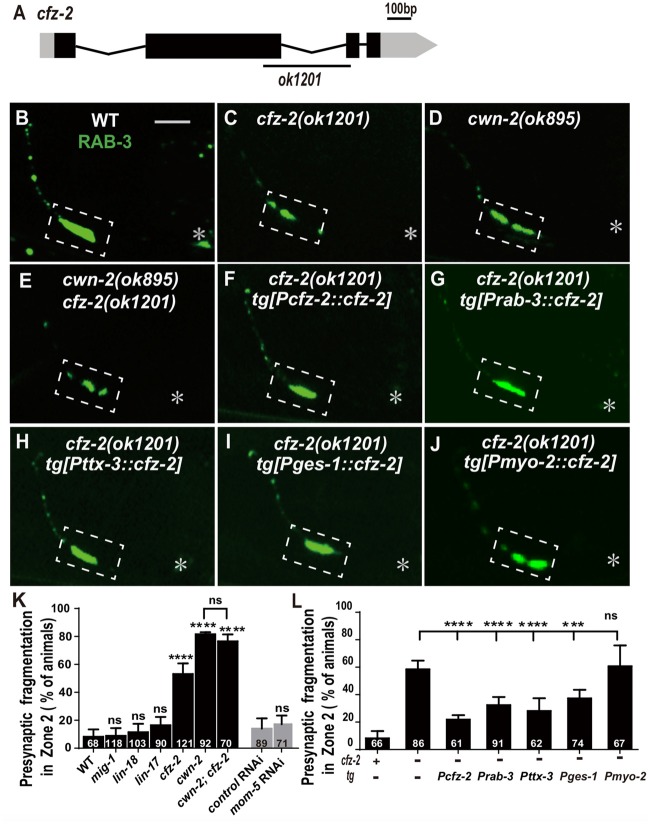
*cfz-2/Fzd* receptor acts with *cwn-2* to promote AIY synaptic clustering. **(A)** A diagram of *cfz-2*. *ok1201* is a 1174 bp deletion allele. The boxes and lines represent exons and introns. Black and gray boxes indicate coding sequence and UTRs, respectively. The line beneath indicates the deletion region. **(B–J)** Confocal micrographs of the AIY presynaptic pattern of GFP::RAB-3. The GFP::RAB-3 forms a large cluster at Zone 2 in wild type animals **(A)**, which is fragmented in *cfz-2(ok1201)* **(B)**, *cwn-2(ok895)* **(C)** and *cwn-2(ok895); cfz-2(ok1201)* double mutants **(D)**. The Zone 2 fragmentation in *cfz-2(ok1201)* is rescued by expressing *cfz-2* with its own promoter **(F)**, pan-neuronal *rab-3* promoter **(G)**, AIY-specific *ttx-3* promoter **(H)**, intestinal-specific *ges-1* **(I)** or pharyngeal-specific *myo-2* promoter **(J)**. Dashed boxes highlight AIY zone 2 and asterisks indicate the position of AIY soma. Scale bar is 10 μm. **(K,L)** Quantification of AIY Zone 2 fragmentation. The penetrance of the fragmentation in *mig-1(e1787), lin-17(n671), cfz-2(ok1201), cwn-2(ok895), cfz-2(ok1201); cwn-2(ok895), mom-5* RNA interference (RNAi) animals **(K)** and tissue specific rescued strains **(L)**. Data indicate that *cfz-2* acts in the *cwn-2* pathway both in the AIY and in the intestine to promote synaptic clustering. ns: not significance, ***p < 0.001, ****p < 0.0001, analyzed by one-way ANOVA Dunnett’s test **(K,L)** and two-tailed Student’s *t*-test **(K)**. Error bars represent 95% confidence interval **(K,L)** and SEM **(K)** respectively.

Wnts bind to FZD receptors and activate downstream cascade signaling pathways. *C. elegans* has four genes encoding FZDs: *mig-1, lin-17, cfz-2* and *mom-5*. To address the requirement of FZDs for AIY synaptic clustering, we examined the AIY synaptic marker GFP::RAB-3 in the loss-of-function mutants *mig-1(e1787), lin-17(n671)* and *cfz-2(ok1201)*. The mutant alleles for *mig-1(e1787), lin-17(n671)* and *cfz-2(ok1201)* are likely to be null since *mig-1(e1787)* and *lin-17(n671)* are nonsense mutations, and *cfz-2(ok1201)* delete 194 predicted amino acids and result in a frameshift (Figure 5A, Supplementary Figures S5A,B; Sawa et al., 1996; Zinovyeva and Forrester, 2005; Pan et al., 2006). The requirement of *mom-5* for AIY synaptic assembly was assayed by RNAi, due to its essential role during development. Significant synaptic fragmentation in AIY are only observed in *cfz-2(ok1201)* mutants (*p* < 0.0001), which can be rescued by wild type *cfz-2* (*P* < 0.0001 none tg vs. tg. Figures 5B,C,F,K, Supplementary Figures S5D–G). To examine if the AIY Zone 2 synaptic clustering defect in *cfz-2(ok1201)* mutants is due to the AIY morphologic defect, we looked the AIY cytoplasmic mCherry and found that the AIY morphology is grossly normal in the *cfz-2(ok1201)* mutants (Supplementary Figure S7).

If CFZ-2 acts as the CWN-2 receptor, the *cfz-2*; *cwn-2* double mutants would phenocopy either *cwn-2(ok895)* or *cfz-2(ok1201)* single mutants. To test this hypothesis, we made *cwn-2(ok895)*; *cfz-2(ok1201)* double mutants and found that the AIY synaptic fragmentation in the double mutants is similar to that in *cwn-2(ok895)* single mutants (*P* = 0.29, Figures 5C–E,K), supporting the hypothesis that CFZ-2 acts as the CWN-2 receptor.

To address where *cfz-2* acts, we examined where *cfz-2* is expressed with transcriptional reporters. We found that *cfz-2* is expressed in the nerve system (including AIY) and intestines (Supplementary Figures S6A–D). Then we expressed *cfz-2* in the nervous system (P*rab-3*), AIY neurons (P*ttx-3*), intestine (P*ges-1*) or pharynx (P*myo-2*), with the endogenous *cfz-2* promoter as a positive control in the *cfz-2(ok1201)* mutants. The AIY fragmentation of *cfz-2(ok1201)* is rescued when *cfz-2* is expressed in the nervous system, AIY, or in the intestine, but not in the pharynx (*P* < 0.0001, 0.0001, 0.001 for P*rab-3::cfz-2*, P*ttx-3::cfz-2* and P*ges-1::cfz-2* respectively, Figures 5G–J,L). These data suggest that *cfz-2* acts both cell-autonomously in AIY and non-cell-autonomously in the intestine to regulate AIY synaptic assembly.

Wnts can also act through the receptor tyrosine kinase (Ryk) to regulate synaptogenesis (Liebl et al., 2008; Lanoue et al., 2017). We tested the requirement of the only *Ryk* homolog *lin-18* (Inoue et al., 2004), for AIY presynaptic assembly. We found that *t* *lin-18(e620)*, a putative null allele (Inoue et al., 2004), did not cause AIY Zone 2 synaptic fragmentation (*P* = 0.28, Figures 5K, Supplementary Figures S5C,H), suggesting that *lin-18/Ryk* is not required for AIY Zone 2 presynaptic clustering.

### DSH-2/DVL and SYS-1/β-Catenin Are Required for AIY Synaptic Clustering

**Figure 6:**
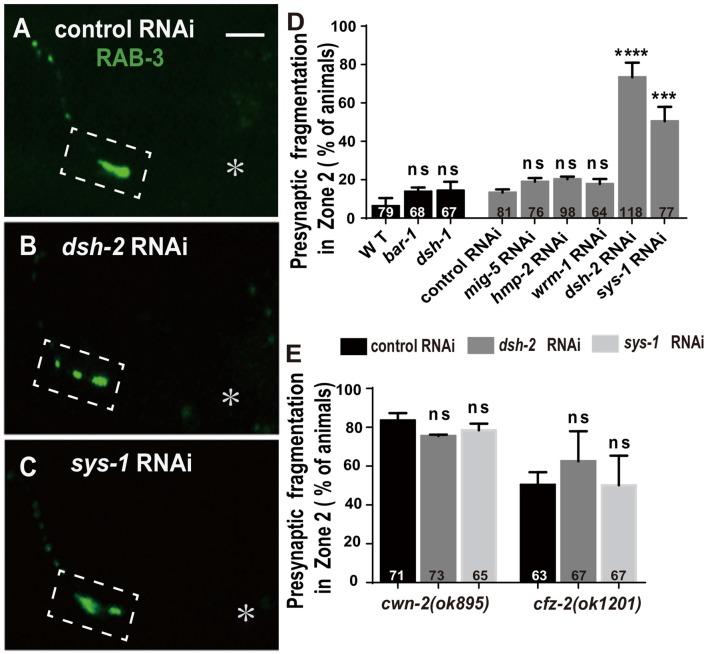
*dsh-2/Dvl* and *sys-1/β-catenin* are required for AIY synaptic clustering.** (A–C)** Confocal micrographs of the AIY presynaptic pattern of GFP::RAB-3 marker in animals treated with control **(A)**, *dsh-2* **(B)** or *sys-1* **(C)** RNAi. The GFP::RAB-3 clustering is normal in control RNAi group, but broken into multiple pieces in *dsh-2* **(B)** or *sys-1* **(C)** RNAi group. Dashed boxes highlight AIY Zone 2 and asterisks indicate the position of AIY soma. Scale bar: 10 μm.** (D,E)** Quantification of AIY Zone 2 fragmentation in *bar-1(mu63), dsh-1(ok1445)* mutants, or *mig-5, hmp-2, wrm-1, dsh-2* and *sys-1* knockdown **(D)**, or knockdown of *dsh-2* or *sys-1* in *cwn-2(ok895)* or *cfz-2(ok1202)* **(E)**. Data indicate that *dsh-2* and *sys-1* are required for AIY presynaptic assembly and act in the *cwn-2/cfz-2* pathway. ns: not significance, ***p < 0.001, ****p < 0.0001 analyzed by one-way ANOVA Dunnett’s test. Error bars represent 95% confidence interval.

The binding of Wnt to FZD receptors activates DVL. Three *C. elegans* DVLs are encoded by: *dsh-1, dsh-2* and *mig-5*. To address if they are required for AIY synaptic clustering, we examined the synaptic marker GFP::RAB-3 in *dsh-1(ok1445)*, a putative hypomorphic allele (Supplementary Figure S8A; Klassen and Shen, 2007), and *dsh-2* and *mig-5* knockdown animals. While we did not observe an AIY synaptic fragmentation in *dsh-1(ok1445)* or *mig-5* knockdown animals, the AIY Zone 2 GFP::RAB-3 morphology in *dsh-2* knockdown animals is similar to that in *cwn-2(ok895)* or *cfz-2(ok1201)* mutants (*p* < 0.0001, Figures 6A,B,D, Supplementary Figures S8C,E,H, and data not shown).

To test whether *dsh-2* acts in the same pathway as *cwn-2* and *cfz-2*, we treated *cwn-2(ok895)* or *cfz-2(ok1201)* mutants with *dsh-2* RNAi. We first determined that the *dsh-2* RNAi efficiency is robust, as assayed by the quantifying synaptic fragmentation of the *dsh-2* RNAi-treated wild type animals (Figure 6D). Interestingly, *dsh-2* RNAi knockdown did not enhance the AIY Zone 2 fragmentation of either *cwn-2(ok895)* or *cfz-2(ok1201)* mutants (*P* = 0.06 for *cwn-2*, *P* = 0.15 for *cfz-2*, Figure 6E), indicating that *dsh-2* acts in the *cwn-2/cfz-2* pathway to promote the synaptic clustering.

In the canonical Wnt pathway, the β-catenin stabilized by DVLs enters nuclei to activate their downstream targets through binding to TCF/LEF. To address if any β-catenin is required for AIY synaptic clustering, we examined the presynaptic marker GFP::RAB-3 in β-catenin knockout or knockdown animals. Four *C. elegans* β-catenin are encoded by *bar-1, hmp-2, wrm-1, sys-1*. While the hypomorphic *bar-1(mu63)* allele (Natarajan et al., 2004, Supplementary Figure S8B), or knockdown of *wrm-1* or *hmp-2* by RNAi does not affect the GFP::RAB-3 clustering in AIY, knockdown of *sys-1* by RNAi results in a significant GFP::RAB-3 fragmentation in AIY Zone 2 (*p* < 0.001, Figures 6C,D, Supplementary Figure S8D,F,G), suggesting that *sys-1* is required for the AIY Zone 2 presynaptic assembly.

To test whether *sys-1* acts in the same pathway as *cwn-2* and *cfz-2*, we treated *cwn-2(ok895)* or *cfz-2(ok1201)* mutants with *sys-1* RNAi. We first determined that the *sys-1* RNAi was efficient as the GFP::RAB-3 fragmentation in the AIY Zone 2 was highly penetrant (Figure 6D). We observed that *sys-1* RNAi did not enhance the AIY synaptic fragmentation of either *cwn-2(ok895)* or *cfz-2(ok1201)* mutants (*P* = 0.43 for *cwn-2*, *P* = 0.99 for *cfz-2* Figure 6E). These results indicate that *sys-1* acts in the *cwn-2/cfz-2* pathway to regulate AIY synaptic clustering.

### POP-1/TCF Is Required for AIY Presynaptic Assembly

**Figure 7:**
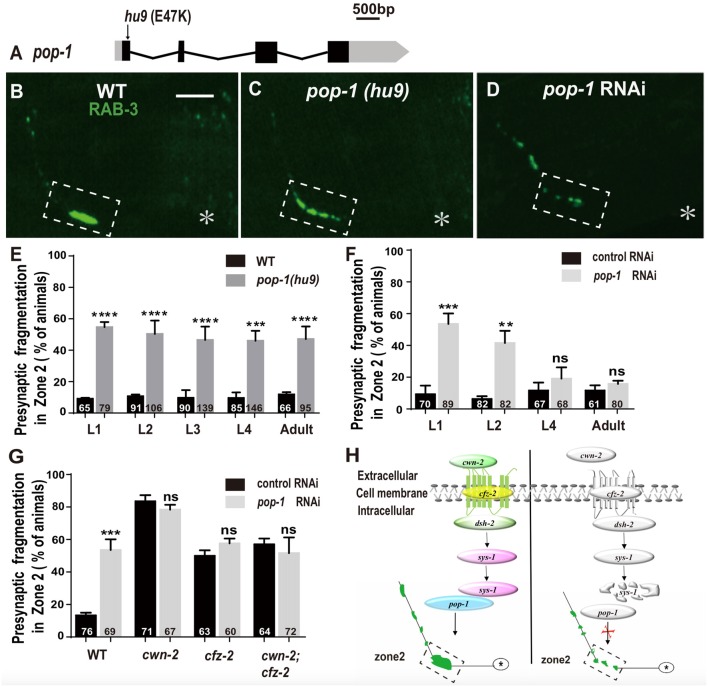
*pop-1/Tcf* is required in Wnt signaling pathway for AIY synaptic clustering. **(A)** A diagram of *pop-1*. *pop-1(hu9)* is a substitution allele. Black and gray boxes indicate coding sequence and UTRs, and lines represent introns. The arrowhead indicates the substitution site. **(B–D)** Confocal micrographs of the AIY presynaptic pattern of GFP::RAB-3 in wild type animals **(B)**, *pop-1(hu9)* mutants **(C)** and *pop-1* RNAi animals **(D)**. The GFP::RAB-3 cluster in the Zone 2 is broken into multiple pieces in *pop-1(hu9)* or *pop-1* RNAi animals **(C,D)**. The dashed boxes highlight the AIY Zone 2. Asterisks indicate AIY soma. Scale bars: 10 μm. **(E–F)** Quantification of the Zone 2 fragmentation of *pop-1(hu9)* mutants at different developmental stages **(E)** and of animals treated with *pop-1* RNAi from L1, L2, L4 or adult stages for 3 days **(F)**. Similar to that in *cwn-2(ok895)* (Figure 3), the fragmentation of Zone 2 emerges at the L1 stage in *pop-1(hu9)* mutants; and knockdown of *pop-1* at L1 or L2, but not L4 or adult stage results robust synaptic fragmentation.** (G)** Quantification of Zone 2 fragmentation in control or *pop-1* RNAi treated wild type, *cwn-2(ok895), cfz-2(ok1021)*, and *cwn-2(ok895); cfz-2(ok1021)* double mutants. Knockdown of *pop-1* resulted in robust AIY Zone 2 fragmentation in wild type animals, but no enhancement in either *cwn-2(ok895), cfz-2(ok1201)* or *cwn-2(ok895); cfz-2(ok1021)* double mutants, suggesting that *cwn-2, cfz-2* and *pop-1* work in the same pathway. n.s.: not significance, **p < 0.01, ***p < 0.001, ****p < 0.0001 by two-tailed Student’s *t* test. Error bars represent SEM. **(H)** A model of regulation of AIY presynaptic assembly by the canonical CWN-2/Wnt pathway. In wild type animals, CWN-2 binds to the Frizzled receptor CFZ-2, by activating the Dishevelled DSH-2 and stabilizing β-catenin SYS-1. The stabilized SYS-1 binds to the TCF/LEF transcription factor POP-1 and promotes AIY synaptic assembly. Animals lose the function of the CWN-2/CFZ-2/DSH-2/SYS-1/POP-1 pathway showing AIY presynaptic fragmentation.

The β-catenin interacts with the TCF transcription factors to activate the expression of downstream targets. *C. elegans* has only one TCF homolog encoded by *pop-1* (Lin et al., 1998). To address the requirement of *pop-1* for AIY synaptic assembly, we first examined the synaptic phenotype in the *pop-1(hu9)* mutants, which harbors a mutation at the β-catenin interaction site (Figure 7A; Korswagen et al., 2002). We found that the AIY Zone 2 fragmentation in *pop-1(hu9)* mutants was similar to that in *cwn-2(ok895)* or *cfz-2(ok1201)* mutants or knockdown of *dsh-2* or *sys-1* animals (Figures 7B,C,E). To further confirm the requirement of *pop-1* for AIY presynaptic assembly, we knocked down *pop-1* by RNAi. We found that *pop-1* knockdown also resulted in a robust AIY Zone 2 fragmentation (Figures 7D,F,G).

To determine if *pop-1* acts in the same pathway as *cwn-2*, first we examined the AIY synaptic phenotype at L1, L2, L3, L4 and adult stages in *pop-1(hu9)* mutants. Similar to *cwn-2(ok895)* mutants, the AIY Zone 2 presynaptic fragmentation appears starting from L1 and continues into adult stage (*P* < 0.0001 for L1–L3 and adult stages, *P* < 0.001 for L4 stage, Figure 7E). Next, we treated wild type animals with *pop-1* RNAi starting at L1, L2, L4 and day 1 adult stages and examined the GFP::RAB-3 in AIY 3 days later. Similar to the results from *cwn-2* RNAi, we observed the synaptic fragmentation in animals treated with *pop-1* RNAi starting at L1 or L2, but not after L4 (*P* < 0.001 for L1, *P* < 0.01 for L2, *P* = 0.21 for L4, and 0.14 for adult stages, Figure 7F), suggesting that *pop-1* is required in larval stages for the presynaptic assembly.

To confirm that *pop-1* acts in the *cwn-2/cfz-2* signal pathway, we knocked down *pop-1* in *cwn-2(ok895)*, *cfz-2(ok1201)*, or *cwn-2(ok895);* *cfz-2(ok1201)* double mutants. We first determined the *pop-1* RNAi efficiency by the penetrance of the AIY Zone 2 presynaptic fragmentation (*P* < 0.001), and embryonic lethality of F1 (data not shown). Supporting our hypothesis, *pop-1* RNAi did not aggravate the expressivity or penetrance of the AIY Zone 2 presynaptic fragmentation of *cwn-2(ok895), cfz-2(ok1201)*, or *cwn-2(ok895); cfz-2(ok1201)* double mutants (*P* = 0.14, 0.13, 0.42 for *cwn-2(ok895), cfz-2(ok1201)*, and *cwn-2(ok895); cfz-2(ok1201)* double mutants, respectively, Figure 7G). These results suggest that *pop-1* acts in the same pathway as *cwn-2/Wnt* and *cfz-2/Frizzled*.

Wnts can also act through non-canonical PCP or Ca2+ signaling pathways, which are mediated by *Vangl* or *CDC42* (Supplementary Figure S1). We examined the effect of deletion alleles *vang-1(ok1142)* and *cdc-42(ok825)* (Supplementary Figures S2D,E) on the AIY synaptic marker GFP::RAB-3. Neither of them shows Zone 2 synaptic fragmentation (Supplementary Figures S2K–M), suggesting that *vang-1* or *cdc-42* is not required for synaptic clustering in AIY Zone 2.

Collectively, our data suggest that CWN-2 functions through the canonical Wnt signal pathway, which requires CFZ-2/FZD, DSH-2/DVL, SYS-1/β-catenin and the only POP-1/TCF to promote AIY presynaptic assembly. This CWN-2/Wnt signaling acts both cell-autonomously in the AIY and non-cell-autonomously in the intestine, both during embryonic and postembryonic development to promote the AIY presynaptic assembly (Figure 7H).

## Discussion

Synapses are key structures for neuronal function, and synaptic assembly is precisely regulated. In this study, we reported a molecular mechanism by which CWN-2/Wnt regulates the presynaptic assembly in interneuron AIY in *C. elegans*. Our results demonstrate that CWN-2 regulates the presynaptic assembly during embryonic and postembryonic development through the canonical Wnt signaling pathway, requiring CFZ-2, DSH-2, and SYS-1, and the TCF transcription factor POP-1.

Our genetic data strongly support that specific components in the canonical Wnt signaling promotes *C. elegans* nerve ring interneuron presynaptic assembly. However, the limitation of this work is that we can not conclusively exclude the requirement of some components in the pathway for two reasons. First, we tested for the requirement of essential genes through RNAi, which could not deplete their expression; second, we scored the synaptic defect mainly based on the fragmentation at Zone 2 region, which will miss those genes that only affect the GFP::RAB-3 intensity or size.

### CWN-2/Wnt Promotes Synaptic Assembly

In this study, we found that *C. elegans* CWN-2 promotes presynaptic assembly as supported by several lines of evidence. First, loss-of-function mutation *cwn-2(ok895)* results in reduction of both synaptic vesicle and active zone markers at AIY presynaptic sites; second, the synaptic defect in *cwn-2(ok895)* is rescued by transforming a wild type copy of *cwn-2*; third, knockdown of *cwn-2* with RNAi decreases the AIY synaptic vesicle GFP::RAB-3 clustering.

*C. elegans* have five Wnts: CWN-1, CWN-2, EGL-20, LIN-44 and MOM-2 (Shackleford et al., 1993; Herman and Horvitz, 1994; Thorpe et al., 1997; Maloof et al., 1999). At presynaptic sites of NMJ, LIN-44 and EGL-20 inhibit synaptic assembly (Klassen and Shen, 2007; Mizumoto and Shen, 2013). The findings that CWN-2 promotes the interneuron AIY presynaptic assembly expand our understanding of the roles of Wnts in *C. elegans* presynaptic assembly. CWN-2 is the closest to Wnt5 in *Drosophila* and mammals (Prud’homme et al., 2002). Similar to the role of CWN-2 in AIY presynaptic assembly, *Drosophila* Wnt5 promotes synaptic formation (Liebl et al., 2008). However, mammalian Wnt5a has a more complex role in hippocampal synaptic development (Davis et al., 2008; Farias et al., 2009; Cuitino et al., 2010; Varela-Nallar et al., 2010; Thakar et al., 2017). Wnt5a was found to promote both glutamatergic spine morphogenesis and GABA receptor trafficking in rat cultured hippocampal neurons (Cuitino et al., 2010; Varela-Nallar et al., 2010), but to inhibit glutamatergic synaptic development in mouse hippocampal neurons (Davis et al., 2008; Thakar et al., 2017). In addition to Wnt5a, other Wnts have been found to either promote or inhibit synaptogenesis in different organisms, suggesting evolutionally conserved roles of Wnts in synaptic development (Budnik and Salinas, 2011; Park and Shen, 2012; Barik et al., 2014). The complex roles of Wnts are partly due to the diversity of Wnts and receptors, various signaling cascades and the complexity and dynamics of synapses in the nervous system. Combined with previous studies (Klassen and Shen, 2007; Mizumoto and Shen, 2013), our findings suggest that like in mammals, *C. elegans* Wnts have both positive and negative roles in regulating presynaptic assembly.

### The POP-1/TCF Mediated Canonical Wnt Pathway Is Required for Presynaptic Assembly

Wnts function through either canonical or noncanonical pathways (Ciani and Salinas, 2005). The canonical pathway is mediated by FZD receptors, DVLs, β-catenin and TCF transcription factors (Wisniewska, 2013). Our study found that CWN-2 promotes presynaptic assembly through the canonical Wnt signaling pathway supported by the following evidence. First, mutation in *cfz-2/Fzd*, or knockdown of *dsh-2/Dvl* or *sys-1/β-catenin* by RNAi mimics the AIY Zone 2 presynaptic fragmentation in either *cwn-2* or *cfz-2* mutants. Second, loss-of-function mutation or knockdown of *pop-1/Tcf* resembles the AIY presynaptic fragmentation observed in either *cwn-2* or *cfz-2* mutants. Third, combination of mutations, or mutations and knockdown of two or three genes described above shows similar degree of the AIY presynaptic fragmentation to that in any single mutants. Collectively, these data suggest that CWN-2 regulates AIY presynaptic assembly through the canonical signal pathway.

In *C. elegans*, at the presynaptic sites, LIN-44 and EGL-20 inhibit synaptic assembly independent of the TCF/POP-1 (Klassen and Shen, 2007; Mizumoto and Shen, 2013). Although mutations of POP-1 enhanced the DD neuron presynaptic assembly defect in FSN-1 mutants, POP-1 single mutants showed normal presynaptic phenotype (Tulgren et al., 2014). In vertebrates or *Drosophila*, the TCF/LEF family of transcription factors can be activated by Wnts (Eastman and Grosschedl, 1999; Korswagen and Clevers, 1999), and is associated with memory consolidation (Fortress et al., 2013), but no evidence indicates its role in synaptic assembly thus far. Our findings showed for the first time that the β-catenin SYS-1 and the TCF transcription factor POP-1 are required for presynaptic assembly in the interneuron AIY.

### Embryonic and Postembryonic Requirement for the Cell-Autonomous and Non-Cell-Autonomous CWN-2 Signal to Promote Synaptic Clustering

CWN-2 is expressed both during embryonic and postembryonic developmental stages, and our data suggests that CWN-2 has a role in synaptic assembly during both stages. First, we found that in *cwn-2(ok895)* or *pop-1(hu9)* mutants, the AIY Zone 2 presynaptic fragmentation appeared in newly hatched L1, suggesting that *cwn-2* and *pop-1* are required during embryonic development. Second, postnatal knockdown of *cwn-2*, *dsh-2*, or *pop-1* with RNAi results in the AIY presynaptic assembly defect, indicating that the Wnt signal is required during larval stages for AIY presynaptic assembly.

AIY presynaptic assembly is largely established during embryonic stages and is maintained throughout the life of the animal (Colón-Ramos et al., 2007; Shao et al., 2013). However, during postembryonic development, as the animal grows, the nervous system architecture, including synaptic structure, scales up (Bénard and Hobert, 2009). We found that while the AIY synaptic distribution is maintained during postnatal development and adult stages, the size or intensity of synaptic marker increases as animals grow (Figure 3). At the NMJs, extracellular matrix (ECM) components such as type IV collagen EMB-9 and ECM remodeling ADAMT proteases such as GON-1 are required for maintaining the synaptic structures during the postnatal stages (Kurshan et al., 2014; Qin et al., 2014). The immunoglobulin superfamily (IgSF) protein ZIG-10 was recently identified for maintaining synaptic densities during development and adulthood (Cherra and Jin, 2016).

Wnts can act either locally or at long distances. Our studies showed that *cwn-2* expressed either in the nervous system (or AIY) or in the intestine rescues the AIY Zone 2 fragmentation in the *cwn-2* loss-of-function mutants, suggesting that both neuronal and intestinal CWN-2/Wnt regulates the AIY presynaptic assembly. However, the presynaptic GFP::RAB-3 intensity can only be rescued by expressing *cwn-2* in the intestine, not in the nerve system, suggesting that the fragmentation effect and the reduction of the GFP::RAB-3 intensity are regulated independently. Alternatively, the fragmentation could be a more severe reduction of the GFP::RAB-3 intensity. Further experiments need to be done to differentiate those possibilities. The data also indicate that CWN-2 from the intestine is probably more important than that from nerve system. Similar to CWN-2, the Frizzled receptor CFZ-2 acts both cell-autonomously and non-cell-autonomously for the fragmentation phenotype. We speculate that the intestinal CWN-2/Wnt signaling is indirect and probably act through secreted signaling molecules. Consistent with this hypothesis, molecules involved in exocytosis or secretion in the intestine are found to regulate neuronal function (Doi and Iwasaki, 2002; Mahoney et al., 2008). Further studies are needed to determine how the CWN-2/Wnt signaling in the intestine regulates synaptogenesis in the AIY neurons.

Wnts are evolutionarily conserved signaling molecules playing critical roles in neural development, including synaptogenesis (Koles and Budnik, 2012; Park and Shen, 2012). Wnt signaling dysfunction is often associated with neurodevelopmental and neurodegenerative disorders such as autism, schizophrenia, bipolar disorder and Alzheimer’s disease (Gould and Manji, 2002; Inestrosa et al., 2012; Kwan et al., 2016). The most common feature for those disorders is the defects of synaptic function. Wnt signaling blockade leads to synaptic disassembly in mature hippocampal neurons and probably some neurodegenerative disorders (Purro et al., 2014). Our finding that the presynaptic development requires the canonical Wnt signal and TCF transcriptional factors might provide cues to develop therapeutic strategies for related neurological disorders.

## Author Contributions

YS and ZS conceived, designed the project. YS, QL and ZS performed experiments and analyzed data, interpreted the results. YS and ZS wrote the manuscript.

## Conflict of Interest Statement

The authors declare that the research was conducted in the absence of any commercial or financial relationships that could be construed as a potential conflict of interest.

## Abbreviations

ADAMT: A disintegrin and metalloproteinase with thrombospondin motifs
AIY: Amphid interneuron
CAMKII: Type II calcium/calmodulin-dependent protein kinase
DVL: Dishevelled
ECM: Extracellular matrix
FZD: Frizzled receptor
GFP: Green fluorescent protein
IgSF: Immunoglobulin superfamily
JNK: c-Jun N-terminal kinase
LEF: Lymphoid enhancing factor
MAP: Microtubule-associated protein
NMJ: Neuromuscular junction
PCP: Planer cell polarity
PK: Prickle
PKC: Protein kinase C
RNAi: RNA interference
TCF: T-cell specific transcription factor.
