# Supplementary material for: Wnts Promote Synaptic Assembly Through T-Cell Specific Transcription Factors in Caenorhabditis elegans
Source: Front Mol Neurosci. 2018 Jun 15;11:194. doi: 10.3389/fnmol.2018.00194 (PMC6013564; doi:10.3389/fnmol.2018.00194)
Supplement: Supplementary file 1 [file Presentation_1.PDF]

Supplementary Material

Wnts promote synaptic assembly through T-cell specific transcription factors in *Caenorhabditis elegans*

Yanjun Shi[1], Qian Li[1], Zhiyong Shao[1*]

1. Department of Neurology, State Key Laboratory of Medical Neurobiology and Institutes of Brain Science, Zhongshan Hospital, Fudan University, Shanghai, P. R. China

* Correspondence:

Dr. Zhiyong Shao

shaozy@fudan.edu.cn

1 Supplementary Figures and Tables

1.1 Supplementary Figures

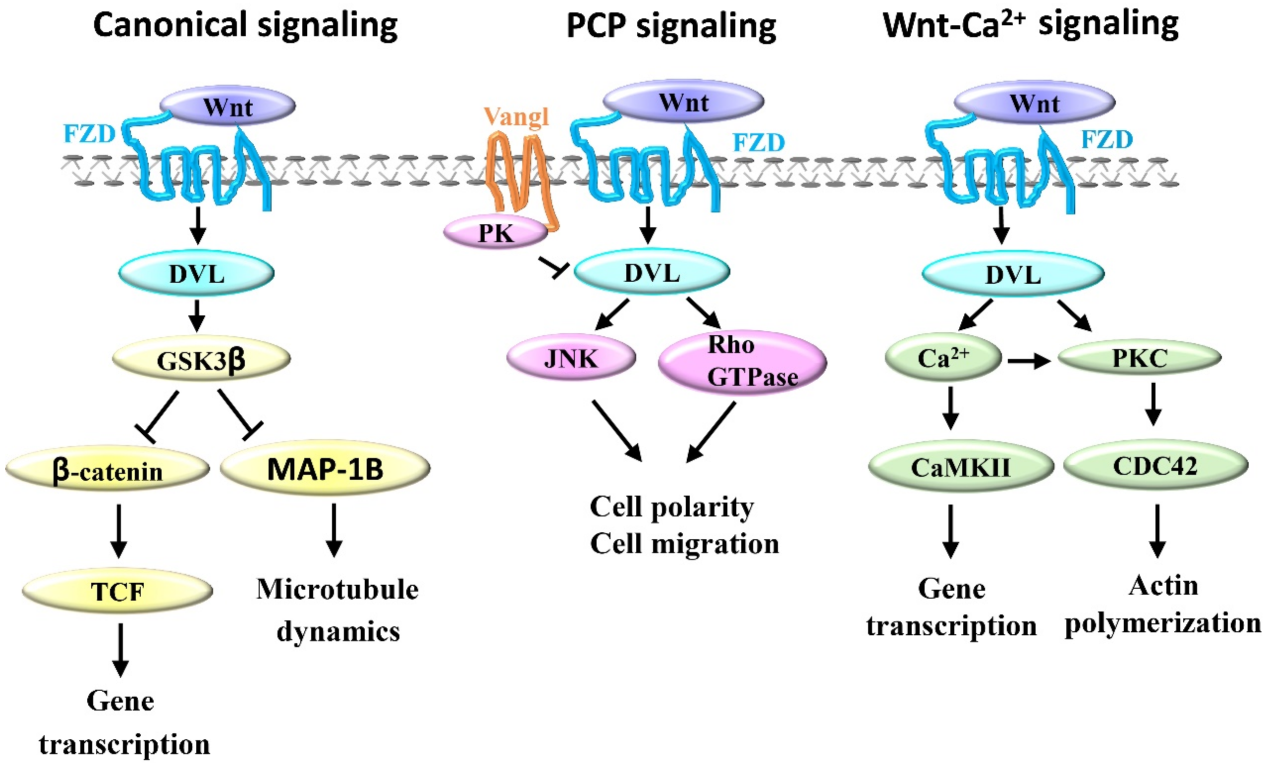

Supplementary Figure 1. Models of Wnt signaling pathways

Wnt ligands activate various signaling pathways to modulate cellular activities. Three major pathways are showed in the diagram. The canonical pathway is mediated through Frizzled receptors (FZD), Dishevelleds (DVL), the GSK3β complex, β-catenin and T-cell specific transcription factors (TCF). The canonical Wnt pathway can also acts through a divergent pathway through microtubule-associated protein (MAP). Two noncanonical Wnt pathways, planer cell polarity (PCP) and Ca^{2+} pathways, share FZ and DVL with the canonical pathway. The PCP pathway activates JNK and Rho GTPase, which is suppressed by Van Gogh(Vangl)/Prickle(PK). The Ca^{2+} pathway acts through protein kinase C (PKC)/CDC42 and type II calcium/calmodulin-dependent protein kinase (CaMKII).

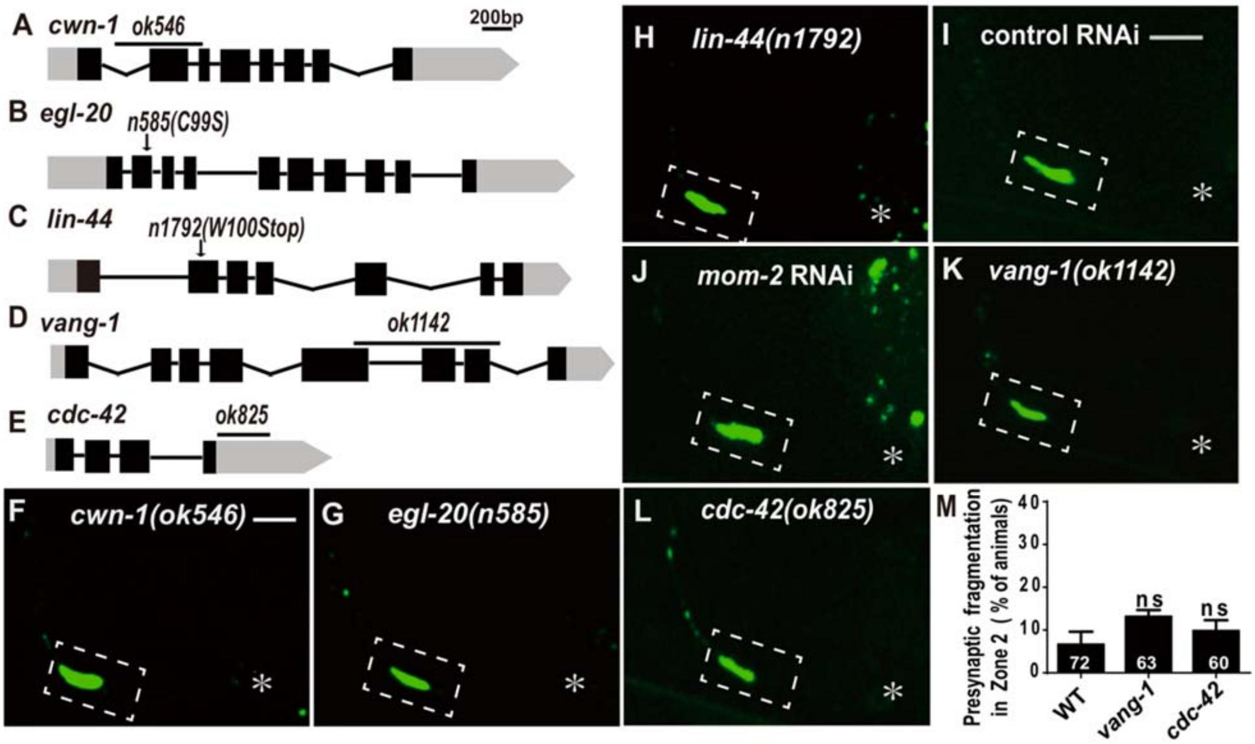

Supplementary Figure 2. CWN-1, EGL-20, LIN-44, MOM-2, VANG-1 and CDC-42 are not required for AIY synaptogenesis

(A-E) Gene structures and mutant alleles of *cwn-1*, *egl-20*, *lin-44*, *vang-1* and *cdc-42*. The boxes and lines represent exons and introns. Black and grey boxes indicate coding sequence and UTRs respectively. The line beneath indicates the deletion region, the arrows indicate substitution sites. **(F-L)** Confocal images of AIY synaptic marker GFP::RAB-3 in *cwn-1(ok546)* (F), *egl-20(n585)* (G), *lin-44(n1792)* (H), RNAi with empty vector control (I) and *mom-2* (J) animals,*vang-1(ok1142)* (K) and *cdc-42(ok825)*(L) mutants. GFP::RAB-3 cluster in AIY zone 2 is normal in *cwn-1(ok546)*, *egl-20(n585)*, *lin-44(n1792)*, *vang-1(ok1142)* and *cdc-42(ok825)* mutants or *mom-2* RNAi knocking down animals. The dashed boxes mark the AIY zone 2. Asterisks indicate the AIY soma. The scale bar is 10μm. **(M)** Quantification of AIY Zone 2 fragmentation ns: not significance indicated groups analyzed by ANOVA. Error bars represent 95% confidence interval. The total number of worms used is showed in the bars. Quantification data for *cwn-1(ok546)*, *egl-20(n585)*, *lin-44(n1792)*, *mom-2* RNAi is shown in Figure 1.

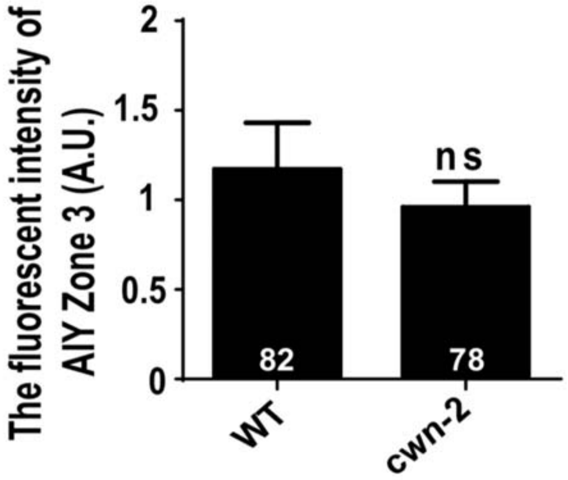

Supplementary Figure 3. Mutation of *cwn-2* does not affect the GFP::RAB-3 intensity of the AIY Zone 3 region

Quantification of AIY Zone 3 (as defined in Figure 1B) GFP::RAB-3 intensity. Data for each genotype are averaged from at least three biological replicates. ns: not significant, analyzed by two-tailed Student's t-test. Error bars represent SEM.

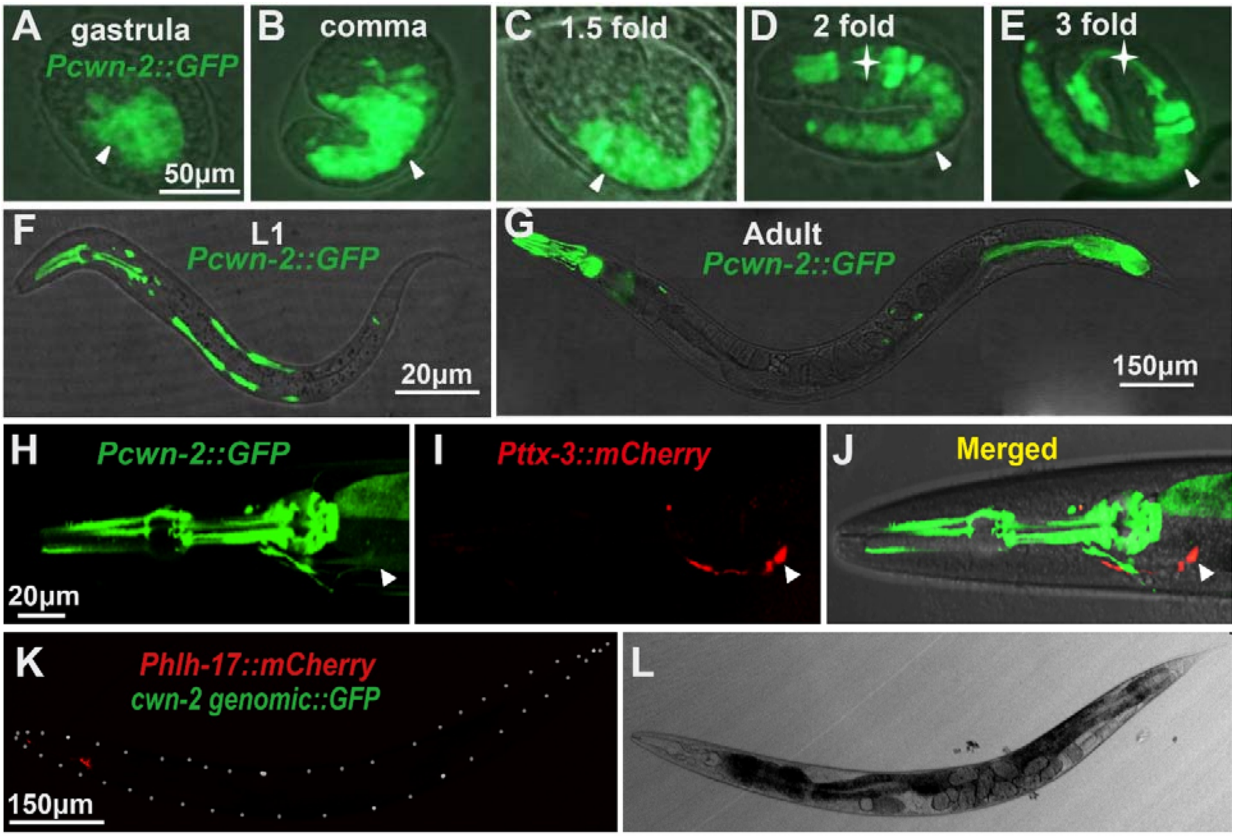

Supplementary Figure 4. ***cwn-2*** **is expressed in pharynx, intestine, body wall muscle and nerve system**

(A-G) Confocal images of embryos or animals with *cwn-2* transcription reporter *Pcwn-2::GFP*. Embryos at gastrula(A), comma (B), 1.5-fold (C), 2-fold (D) and 3-fold (E), and animals at L1 (F) and adult (G) stages. (A-E) are merged bright and GFP channels. Before the 2-fold stage, the transcription reporter *Pcwn-2::GFP* is mainly expressed in the intestine (arrowhead, A-C). Starting from the 2-fold stage, the reporter is dramatically increased in the pharyngeal bulb (D as indicated by stars). After hatching, *Pcwn-2::GFP* is seen in the pharynx, intestine, body wall muscles and some neurons, which is represented by L1 stage (F) and adult stage (G). **(H-J)** Confocal images of *Pcwn-2::GFP* and *Pttx-3::mCherry* in the adult head with green (H), red (I) and green, red and white field (J). The *Pcwn-2::GFP* is not expressed in the AIY. **(K-L)** Micrographs of *cwn-2genomic::GFP* with green and red channel (K) or white field (L). No GFP is seen in the animal (in the dashed boxes, which indicates that the *cwn-2* genomic sequence does not have promoter elements.

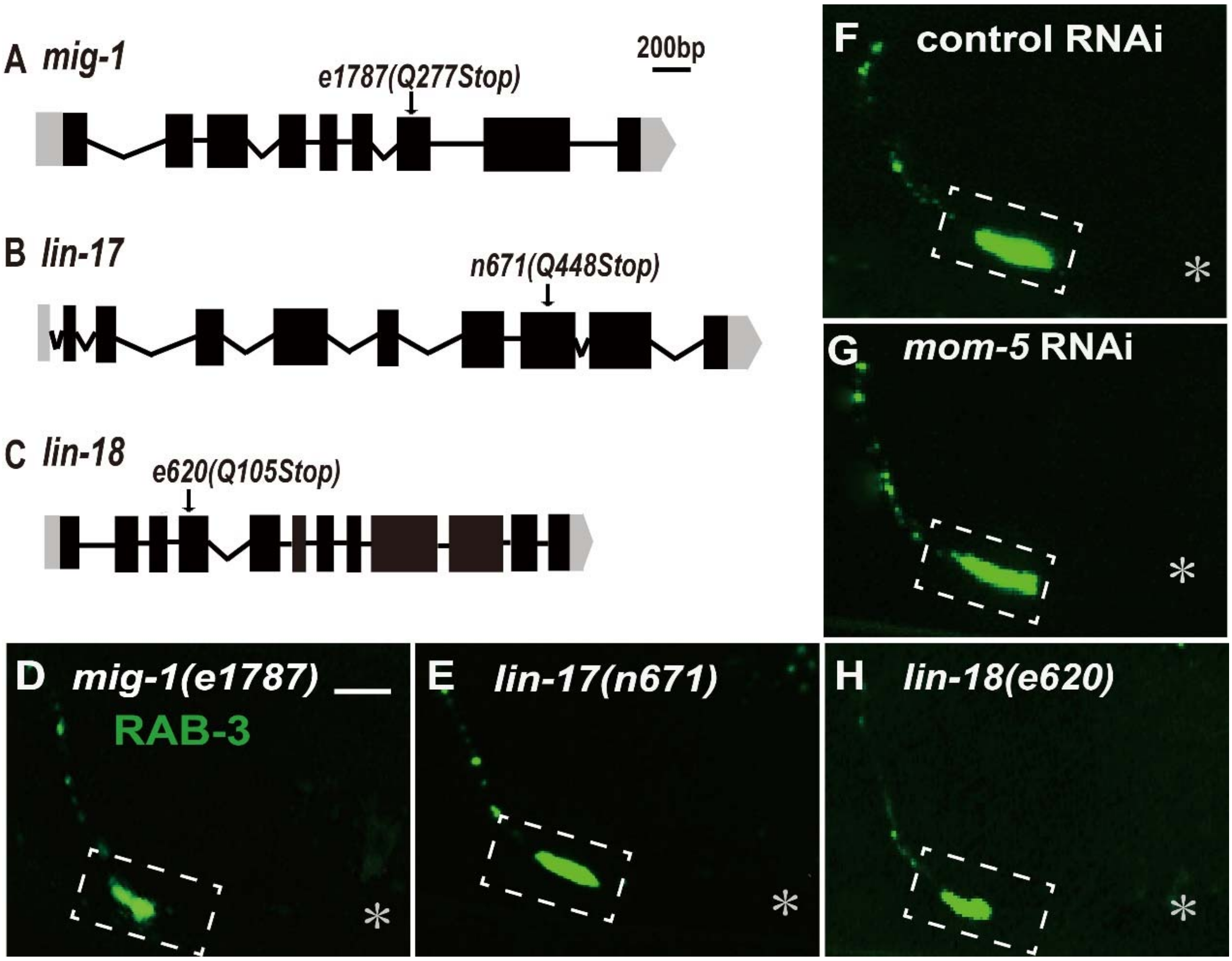

Supplementary Figure 5. Frizzled receptors encoding by *mig-1*, *lin-17*, *mom-5* and the receptor tyrosine kinase (RYK) encoding by *lin-18* are not required for AIY presynaptic assembly

(A-C) Gene structures and mutant alleles of *mig-1*, *lin-17* and *lin-18* mutants. The boxes and lines represent exons and introns. Black and grey boxes indicate coding sequence and UTRs. The arrows indicate the substitution sites. **(D-H)** Confocal micrographs of AIY presynaptic GFP::RAB-3 in *mig-1(e1787)* (D), *lin-17(n671)* (E), *lin-18(e620)* (H) mutants, and control (F) and *mom-5* RNAi (G) treated animals. AIY synaptic structure is not affected in *mig-1*(e1787), *lin-17(n671)*, *lin-18(e620)* mutants and *mom-5* RNAi animals. The dashed boxes mark the AIY zone 2. Asterisks indicate the AIY soma. The scale bar is 10µm. Quantification data is shown in Figure 5.

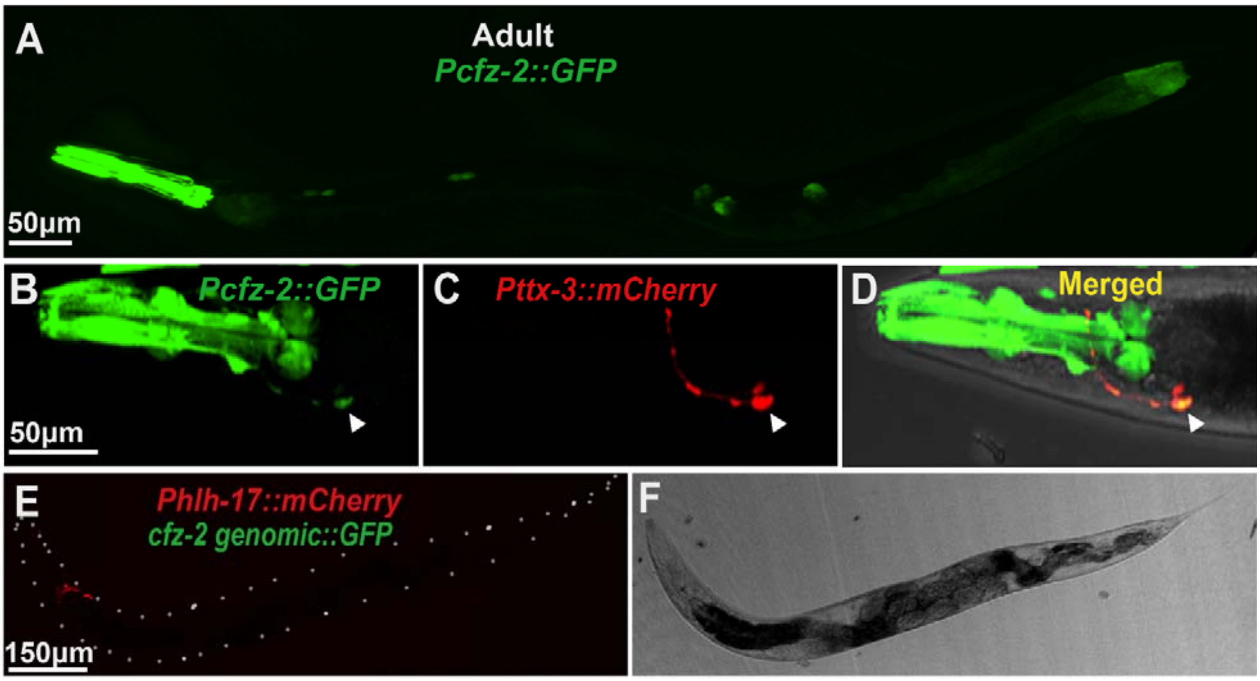

Supplementary Figure 6. The *cfz-2* is expressed in the pharynx, intestine and nerve system including AIY interneurons

(A) Micrographs of *Pcfz-2::GFP* expression in an adult animal. **(B-D)** Micrographs of an adult head labelled with *Pcfz-2::GFP* and the AIY specific marker *Pttx-3::mCherry* with green channel (B), red channel (C) and green, red and white channels (D). As indicated by the arrow head, *Pcfz-2::GFP* and *Pttx-3::mCherry* colabel the AIY. **(E-F)** Micrographs of *cfz-2genomic::GFP* with *Phlh-17::mCherry* as a coinjection marker with green and red channel (E) and bright field channel (F). No GFP is visible in *cfz-2genomic::GFP* transgenic animals, which suggests that *cfz-2genomic* does not have promoter regulatory elements.

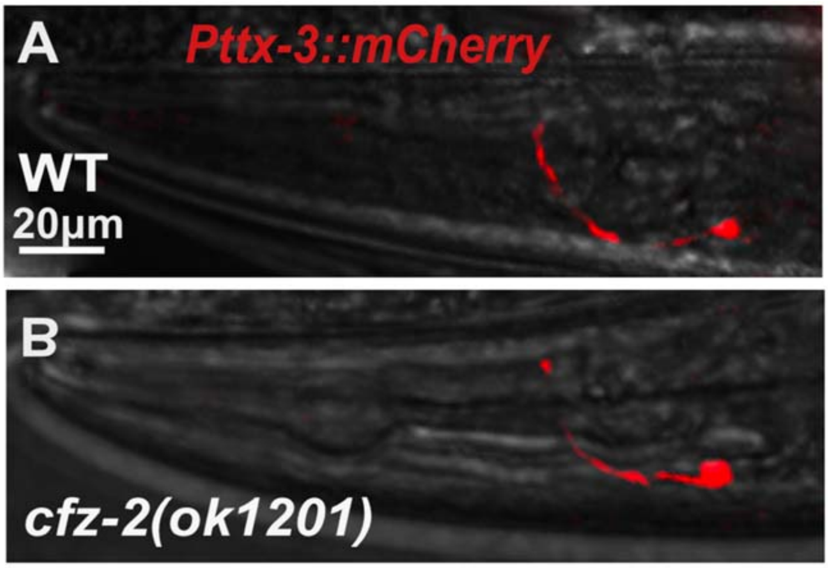

Supplementary Figure 7. Mutation of *cfz-2* does not affect the AIY gross morphology

(A-B) Micrographs of AIY cytoplasmic mCherry (red) merged with white field channel in wild type (A) and *cfz-2(ok1201)* mutants. The AIY gross morphology is normal in the *cfz-2(ok1201)* mutants.

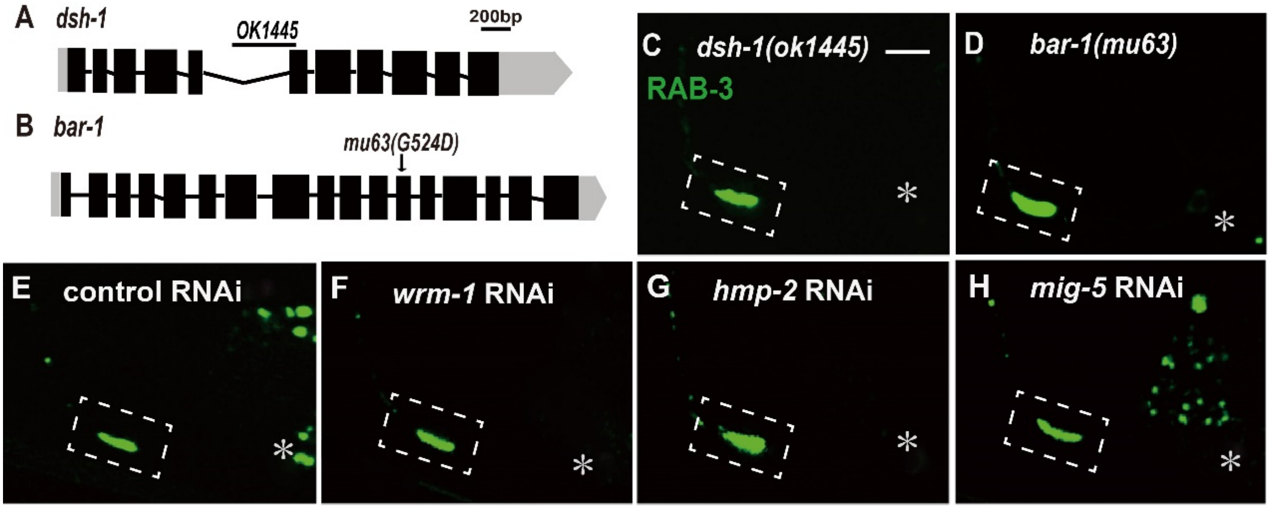

Supplementary Figure 8. The Dishevelled DSH-1 and MIG-5, the β-catenin BAR-1, WRM-1, and HMP-2 are not required for AIY presynaptic clustering

(A-B) Gene structures and mutant alleles of *dsh-1* and *bar-1*. The boxes and lines represent exons and introns. Black and grey boxes indicate coding sequence and UTRs. The line beneath indicates the deletion region, the arrow indicates the substitution site. **(C-H)** AIY presynaptic GFP::RAB-3 in *dsh-1(ok1445)* (C), *bar-1(mu63)* (D) mutants, and control (E), *wrm-1* (F), *hmp-2* (G) and *mig-5* (H) RNAi treated animals. AIY synaptic assembly is not affected in *dsh-1(ok1445)*, *bar-1(mu63)* mutants and *wrm-1*, *hmp-2* and *mig-5* RNAi knocking down animals. The dashed boxes mark the AIY zone 2. Asterisks indicate the AIY soma. The scale bar is 10μm. Quantification data is shown in Figure 6.

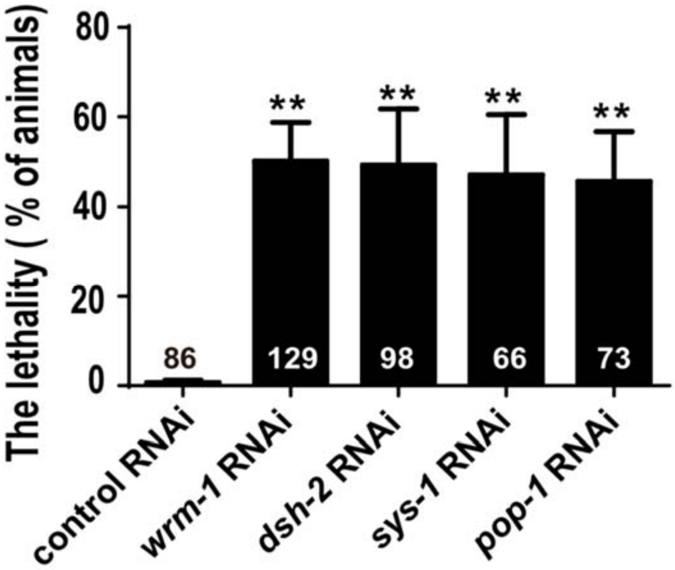

Supplementary Figure 9. RNAi efficiency as assayed by quantifying the percentage of dead animals

Quantification of percentage of dead animals at the adult (day 1) stage caused RNAi knockdown of *wrm-1, dsh-2, sys-1* and *pop-1*. Data for each genotype are averaged from at least three biological replicates. **: P<0.01 analyzed by one-way ANOVA, error bars represent 95% confidence interval.

Supplementary Tables

Supplementary Table 1. Strains used in this study

Strain Name	Genotype
N2	Wild type
TV392	*wyls45 (Pttx-3::GFP::rab-3, Punc-122::RFP)*
FDU163	*cwn-1(ok546); wyls45(Pttx-3::GFP::rab-3, Punc-122::RFP)*
FDU116	*cwn-2(ok895); wyls45(Pttx-3::GFP::rab-3, Punc-122::RFP)*
FDU229	*lin-44(n1792); wyls45(Pttx-3::GFP::rab-3, Punc-122::RFP)*
FDU224	*egl-20(n585); wyls45(Pttx-3::GFP::rab-3, Punc-122::RFP)*
FDU775	*olais10(Pttx-3::GFP::syd-1, Pttx-3::mcherry::rab-3, Punc-122 ::RFP)*
FDU966	*cwn-2; olaIs10(Pttx-3::GFP::syd-1, Pttx-3::mcherry::rab-3, Punc-122 ::RFP)*
FDU358	*mig-1(e1787); wyIs45(Pttx-3::GFP::rab-3, Punc-122::RFP)*
FDU357	*lin-18(e620); wyIs45(Pttx-3::GFP::rab-3, Punc-122::RFP)*
DCR1659	*lin-17(n671); wyIs45(Pttx-3::GFP::rab-3, Punc-122::RFP)*
FDU503	*cfz-2(ok1201); wyIs45(Pttx-3::GFP::rab-3, Punc-122::RFP)*
FDU502	*cwn-2(ok895); cfz-2(ok1201); wyls45(Pttx-3::GFP::rab-3, Punc-122::RFP)*
FDU930	*vang-1(ok1142); wyIs45(Pttx-3::GFP::rab-3, Punc-122::RFP)*
FDU931	*cdc-42(ok825); wyIs45(Pttx-3::GFP::rab-3, Punc-122::RFP)*
FDU1165	*pop-1(hu9); wyIs45(Pttx-3::GFP::rab-3, Punc-122::RFP)*

Continued supplementary Table 1 (Strains used in this study)

FDU86	*dsh-1(ok1445); wyIs45(Pttx-3::GFP::rab-3, Punc-122::RFP)*
FDU355	*bar-1(mu63); wyIs45(Pttx-3::GFP::rab-3, Punc-122::RFP)*
FDU568	*shcEx293 (Pcwn-2::gfp, Punc-122::RFP)*
FDU692	*shcEx312 (Pcwn-2::gfp, Punc-122::RFP)*
FDU222	*cwn-2(ok895); wyls45 (Pttx-3::GFP::rab-3, Punc-122::RFP)X; shcEx112 (Pcwn-2::cwn-2, Punc-122::GFP)*
FDU225	*cwn-2(ok895); wyls45 (Pttx-3::GFP::rab-3, Punc-122::RFP)X; shcEx113 (Pcwn-2::cwn-2, Punc-122::GFP)*
FDU226	*cwn-2(ok895); wyls45 (Pttx-3::GFP::rab-3, Punc-122::RFP)X; shcEx114 (Pcwn-2::cwn-2, Punc-122::GFP)*
FDU969	*cwn-2(ok895); wyls45 (Pttx-3::GFP::rab-3, Punc-122::RFP)X; shcEx437 (Prab-3::cwn-2, Punc-122::GFP)*
FDU970	*cwn-2(ok895); wyls45 (Pttx-3::GFP::rab-3, Punc-122::RFP)X; shcEx438 (Prab-3::cwn-2, Punc-122::GFP)*
FDU587	*cwn-2(ok895); wyls45 (Pttx-3::GFP::rab-3, Punc-122::RFP)X; shcEx280 (Prab-3::cwn-2, Punc-122::GFP)*
FDU562	*cwn-2(ok895); wyls45 (Pttx-3::GFP::rab-3, Punc-122::RFP)X; shcEx267 (Pmyo-2::cwn-2, Punc-122::GFP)*
FDU586	*cwn-2(ok895); wyls45 (Pttx-3::GFP::rab-3, Punc-122::RFP)X; shcEx279 (Pmyo-2::cwn-2, Phlh-17::mcherry)*
FDU971	*cwn-2(ok895); wyls45 (Pttx-3::GFP::rab-3, Punc-122::RFP)X; shcEx439 (Pmyo-3::cwn-2, Punc-122::GFP)*

Continued supplementary Table 1 (Strains used in this study)

FDU972	*cwn-2(ok895); wyls45 (Pttx-3::GFP::rab-3, Punc-122::RFP)X; shcEx440 (Pmyo-3::cwn-2, Punc-122::GFP)*
FDU1109	*cwn-2(ok895); wyls45 (Pttx-3::GFP::rab-3, Punc-122::RFP)X; shcEx448 (Pttx-3::cwn-2, Punc-122::GFP)*
FDU1110	*cwn-2(ok895); wyls45 (Pttx-3::GFP::rab-3, Punc-122::RFP)X; shcEx449 (Pttx-3::cwn-2, Punc-122::GFP)*
FDU1111	*cwn-2(ok895); wyls45 (Pttx-3::GFP::rab-3, Punc-122::RFP)X; shcEx450 (Pges-1::cwn-2, Punc-122::GFP)*
FDU1112	*cwn-2(ok895); wyls45 (Pttx-3::GFP::rab-3, Punc-122::RFP)X; shcEx451 (Pges-1::cwn-2, Punc-122::GFP)*
FDU1113	*cwn-2(ok895); wyls45 (Pttx-3::GFP::rab-3, Punc-122::RFP)X; shcEx452 (Pges-1::cwn-2, Punc-122::GFP)*
FDU1166	*cfz-2(ok1201); wyls45 (Pttx-3::GFP::rab-3, Punc-122::RFP)X; shcEx484 (Pcfz::cfz-2, Punc-122::GFP)*
FDU1167	*cfz-2(ok1201); wyls45 (Pttx-3::GFP::rab-3, Punc-122::RFP)X; shcEx485 (Pcfz::cfz-2, Punc-122::GFP)*
FDU1033	*cfz-2(ok1201); wyls45 (Pttx-3::GFP::rab-3, Punc-122::RFP)X; shcEx444 (Pttx-3::cfz-2, Punc-122::GFP)*
FDU1034	*cfz-2(ok1201); wyls45 (Pttx-3::GFP::rab-3, Punc-122::RFP)X; shcEx445 (Pttx-3::cfz-2, Punc-122::GFP)*
FDU1035	*cfz-2(ok1201); wyls45 (Pttx-3::GFP::rab-3, Punc-122::RFP)X; shcEx446 (Pttx-3::cfz-2, Punc-122::GFP)*
FDU1160	*cfz-2(ok1201); wyls45 (Pttx-3::GFP::rab-3, Punc-122::RFP)X; shcEx479 (Pges-1::cfz-2, Punc-122::GFP)*

Continued Supplementary Table 1 (Strains used in this study)

FDU1161	*cfz-2(ok1201); wyls45 (Pttx-3::GFP::rab-3, Punc-122::RFP)X; shcEx480 (Pges-1::cfz-2, Punc-122::GFP)*
FDU1162	*cfz-2(ok1201); wyls45 (Pttx-3::GFP::rab-3, Punc-122::RFP)X; shcEx481 (Pges-1::cfz-2, Punc-122::GFP)*
FDU1115	*cfz-2(ok1201); wyls45 (Pttx-3::GFP::rab-3, Punc-122::RFP)X; shcEx453 (Prab-3::cfz-2, Punc-122::GFP)*
FDU1116	*cfz-2(ok1201); wyls45 (Pttx-3::GFP::rab-3, Punc-122::RFP)X; shcEx454 (Prab-3::cfz-2, Punc-122::GFP)*
FDU1117	*cfz-2(ok1201); wyls45 (Pttx-3::GFP::rab-3, Punc-122::RFP)X; shcEx455 (Prab-3::cfz-2, Punc-122::GFP)*
FDU1163	*cfz-2(ok1201); wyls45 (Pttx-3::GFP::rab-3, Punc-122::RFP)X; shcEx482 (Pmyo-2::cfz-2, Punc-122::GFP)*
FDU1164	*cfz-2(ok1201); wyls45 (Pttx-3::GFP::rab-3, Punc-122::RFP)X; shcEx483 (Pmyo-2::cfz-2, Punc-122::GFP)*

Table S2. Constructs used in this study

**Construct Name**	**Vector**	**Comments/Reference**
Pcwn-2::sl2::gfp	pDEST	The Gateway system was described in (Norlia Basherudin et al., 2006)
Prab-3::cwn-2[1]	pDEST	*Prab-3* was described in (Nonet et al., 1997)
Pmyo-3::cwn-2[1]	pDEST	*Pmyo-3* was described in (Okkema et al., 1993)
Pmyo-2::cwn-2[1]	pDEST	*Pmyo-2* was described in (Okkema et al., 1993)
Pttx-3::sl2::cwn-2[1]	pSM	*Pttx-3g* was described in (Wenick and Hobert, 2004)
Pges-1::cwn-2[1]	pSM	*Pges-1* was described in (McGhee JD et al., 1990)
Pcfz-2::cfz-2[1]	pSM	The *cfz-2* promoter was 2.9kb upstream the start codon
Prab-3:: cfz-2[1]	pDEST	*Prab-3* was described in (Nonet et al., 1997)
Pmyo-2:: cfz-2[1]	pDEST	*Pmyo-2* was described in (Okkema et al., 1993)
Pges-1::cfz-2[1]	pDEST	*Pges-1* was described in (McGhee JD et al., 1990)
Pttx-3::sl2::cfz-2[1]	pSM	*Pttx-3g* was described in (Wenick and Hobert, 2004)
mom-2 RNAi	pPD129.36[2]	The full length of *mom-2 cDNA*
mom-5 RNAi	pPD129.36[2]	The *mom-5 cDNA* was 1.7kb overlapping region of a, b isoforms

Continued Supplementary Table 1 (Constructs used in this study)

dsh-2 RNAi	pPD129.36[2]	The full length of *dsh-2 cDNA*
mig-5 RNAi	pPD129.36[2]	2kb *mig-5 cDNA* sequence in the common region of a, b, c isoforms
wrm-1 RNAi		From Y Shen (personal communication)
hmp-2 RNAi		From Y Shen (personal communication)
sys-1 RNAi	pPD129.36[2]	2.7kb *sys-1* genomic sequence in the common region of a, b, c isoforms

Note: 1. The coding region of *cwn-2* and *cfz-2* is from the corresponding genomic sequence. 2. pPD129.36: L4440
